# YOLOv8-FDA: lightweight wheat ear detection and counting in drone images based on improved YOLOv8

**DOI:** 10.3389/fpls.2025.1682243

**Published:** 2025-09-24

**Authors:** Yuxuan Lin, Xiao Xiao, Haifeng Lin

**Affiliations:** College of Information Science and Technology, Nanjing Forestry University, Nanjing, China

**Keywords:** wheat ear detection and counting, deep learning, lightweight, HWD, YOLOv8-FDA

## Abstract

**Introduction:**

Wheat is a vital global staple crop, where accurate ear detection and counting are essential for yield prediction and field management. However, the complexity of field environments poses significant challenges to achieving lightweight yet high-precision detection.

**Methods:**

This study proposes YOLOv8-FDA, a lightweight detection and counting method based on YOLOv8. The approach integrates RFAConv for enhanced feature extraction, DySample for efficient multi-scale upsampling, HWD for compressed and accelerated model training, and the SDL loss for improved bounding box regression.

**Results:**

Experimental results on the GWHD dataset show that YOLOv8-FDA achieves a precision of 86.3%, recall of 77.5%, and mAP@0.5 of 84.9%, outperforming the original YOLOv8n by significant margins. The model size is 2.96MB with a computational cost of 8.3 GFLOPs, and it operates at 19.2 FPS, enabling real-time counting with over 97.5% accuracy using cross-row segmentation.

**Discussion:**

The proposed YOLOv8-FDA model demonstrates strong detection performance, lightweight characteristics, and efficient real-time capability, indicating its high practicality and suitability for deployment in real-world agricultural applications.

## 1 Introduction

Wheat is a core staple crop for global food security, with its planted area, production, and trade volume ranking first among all types of food crops (noa, 2025). Ensuring the sustainable development of the wheat industry is therefore crucial to maintaining long-term global food stability. Wheat yield directly impacts human survival and social development. The number of wheat heads in the field, as a key indicator for accurate yield prediction (Maji et al., 2022), plays a vital role in wheat yield estimation, breeding, cultivation management, and phenotypic analysis. High-throughput detection and quantification of wheat heads is essential for assessing wheat growth and density. Therefore, research on wheat detection and counting holds substantial significance. However, counting wheat heads from drone images in real field environments remains challenging due to large image sizes, object size calibration, dense object distribution, and instance overlap.

Traditional detection and counting of wheat heads depend on manual labor, which consumes a great deal of resources and is subjective (Li et al., 2024a). As machine learning and deep learning technologies are on the rise, real-time counting methods have become a research hotspot. Currently, there are three main methods for wheat head detection and counting: image processing (IP), machine learning (ML), and deep learning (DL). In IP-based research, Fernandez-Gallego et al. (2020) utilized RGB images obtained from Unmanned Aerial Vehicles to obtain the number of wheat heads in field images by filtering and locating local peaks, achieving a detection accuracy of 90%. In ML-based research, (Carlier et al., 2022) proposed a multi-sensor fusion classification method based on RGB and multispectral superpixel features, achieving a spike detection accuracy a of 94% using SVM (Hearst et al., 1998), but the method suffers from poor real-time performance. In past few years, DL-based algorithms for object detection have been applied more frequently to detect and count wheat heads. These methods mainly include two-stage algorithms represented by the R-CNN (Girshick et al., 2014) series, which first generates a series of candidate boxes, followed by object classification and location refinement, and single-stage algorithms typified by the YOLO (Redmon et al., 2016) and SSD (Liu et al., 2016) series, which bypass candidate box generation by directly formulating object localization as a regression problem. In terms of two-stage methods, Li et al. (2022b) adopted the Faster R-CNN to achieve wheat head image detection and site localization. The study confirmed that the spike number (SN) prediction model performed robustly in the validation of the manually labeled dataset (MSN), with an average accuracy of 86.7%. In terms of single-stage detectors, Khaki et al. (2022) designed WheatNet, which uses a lightweight pruned MobileNetV2 (Sandler et al., 2018) as the core feature extractor, achieving high accuracy in wheat head detection and counting with real-time processing capabilities, thereby directly serving field decision-making systems. (Li and Wu, 2022) proposed an enhanced YOLOv5 architecture, which significantly improves small wheat head detection accuracy through data augmentation optimization, feature pyramid reconstruction, and dual attention mechanism. The improved model achieved 94.3% mAP on the test set, effectively overcoming issues of missed detection and misidentification of dense wheat heads. Although single-stage and two-stage detectors obtain comparable accuracy in wheat head detection, single-stage methods are remarkably better suited for real-time field counting tasks owing to their swifter inference speed and reduced computational overhead.

In modern agriculture, YOLO-based detectors have become indispensable for real-time monitoring because of their high precision and inference speed. Shen et al. (2023a) put forward an improved YOLOv5 algorithm using separable convolution to replace standard convolution combined with a co-attention mechanism, which improves the accuracy of detection in intricate large-scale field backgrounds with overlap and occlusion. However, the algorithm’s ability to detect wheat spikes at the image edges, where they are not fully displayed, is inadequate. Li et al. (2024b) integrated DCNv3 (Wang et al., 2023b), PConv (Chen et al., 2023) and BiFPN (Tan et al., 2020) to reconstruct the detection architecture, and introduced a CBAM (Jy and Kweon, 2018) to enhance feature fusion. They proposed the YOLOv7-DeepSORT variant with deep compression, achieving a significant reduction in model size while simultaneously improving detection accuracy. (Shen et al., 2023b), on the other hand, developed the ultra-small S-YOLOv5s model based on ShuffleNetV2’s (Gong, 2024) channel compression strategy, combined with lightweight upsampling feature reconstruction technology, maintaining superior detection performance even under extreme parameter compression conditions. The current research focus is on reducing the model parameter size while improving the accuracy of wheat head detection.

In summary, compared to traditional manual calculations, IP and ML methods save labor and resources, and reduce the impact of subjective factors. However, these methods are generally complex and highly dependent on image features such as color and texture, resulting in poor robustness. DL-based methods, on the other hand, overcome the reliance on image-related features and made significant progress in detection performance under complex environments. However, these methods are not well-suited for detecting small wheat head targets, resulting in a substantial decline in accuracy. Moreover, most existing models contain a large number of parameters, limiting the lightweight deployment in real-world field environments. To tackle these problems, this paper puts forward an enhanced lightweight YOLOv8 model that maintains robust detection performance even in complex environments for small-sized wheat heads. The core contributions of this paper are summarized as follows:

1. The use of the RFAConv module enhances feature extraction capabilities for wheat heads, reduces redundant computations and memory access, and improves spatial feature capture.
2. The introduction of the Dysample module enables dynamic adjustment of feature map scales, optimizing computational resource utilization and enhancing the preservation of detailed wheat head information.
3. The adoption of HWD enables more efficient downsampling, reducing the number of parameters while preserving frequency-domain features, thus improving detection efficiency.
4. The integration of the SDL loss function jointly optimizes the distance and shape fitting of bounding boxes, remarkably improving the accuracy of wheat head detection and counting and expediting model convergence.

## 2 Materials and methods

### 2.1 Dataset preparation

**Figure 1:**
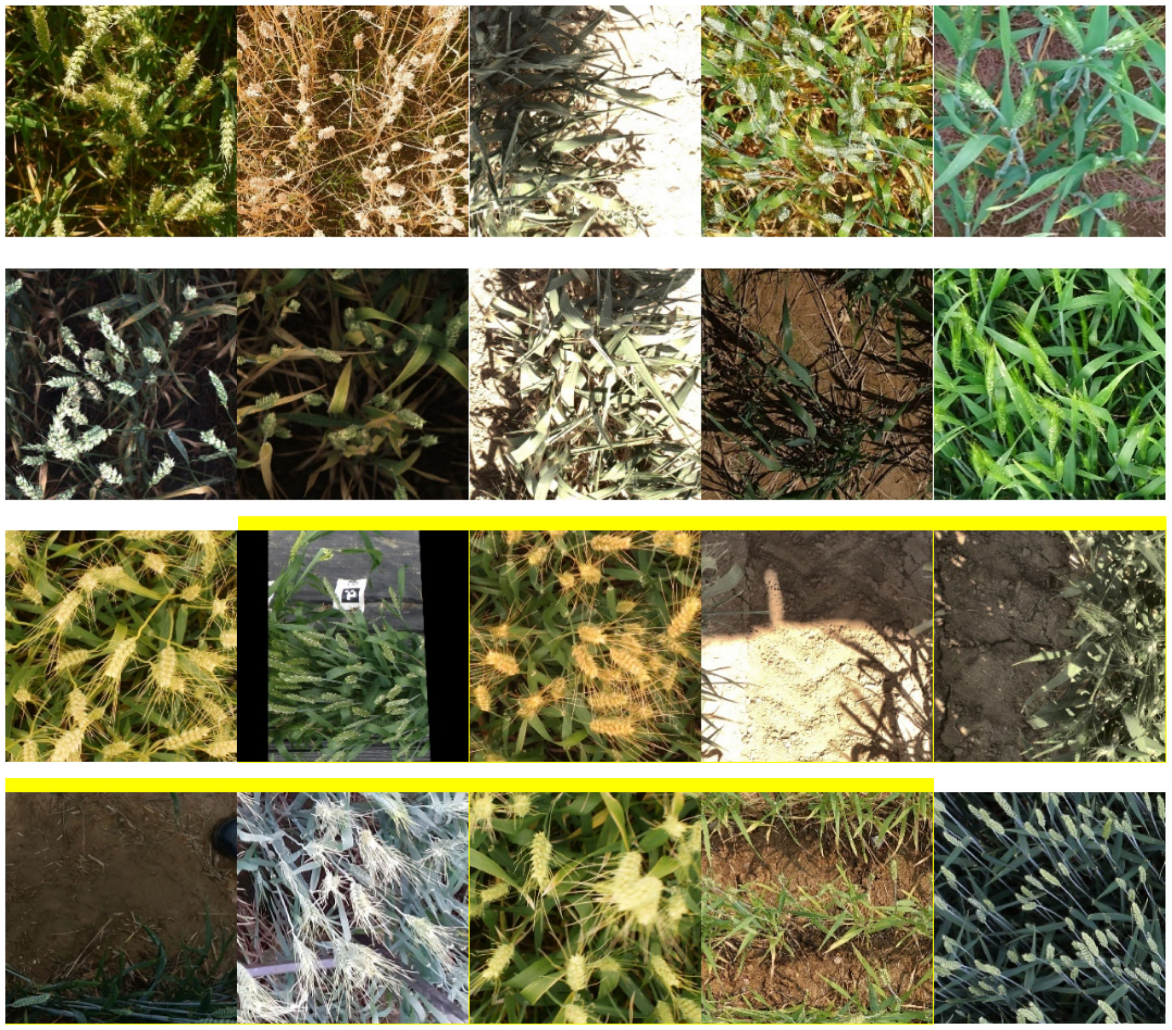
GWHD dataset example.

This study uses the 2021 GWHD (David et al., 2021) public dataset, which contains over 6,000 wheat spike images from 16 research institutions across 12 countries, with a resolution of 1024 × 1024. This dataset covers diverse wheat varieties, planting densities, growth stages, field conditions, and acquisition methods, as illustrated in **Figure 1**. The dataset was divided into training, validation, and test sets in an 8:1:1 ratio.

### 2.2 Enhancement of the YOLOv8 model

#### 2.2.1 Fundamental network architecture of the YOLOv8 model

**Figure 2:**
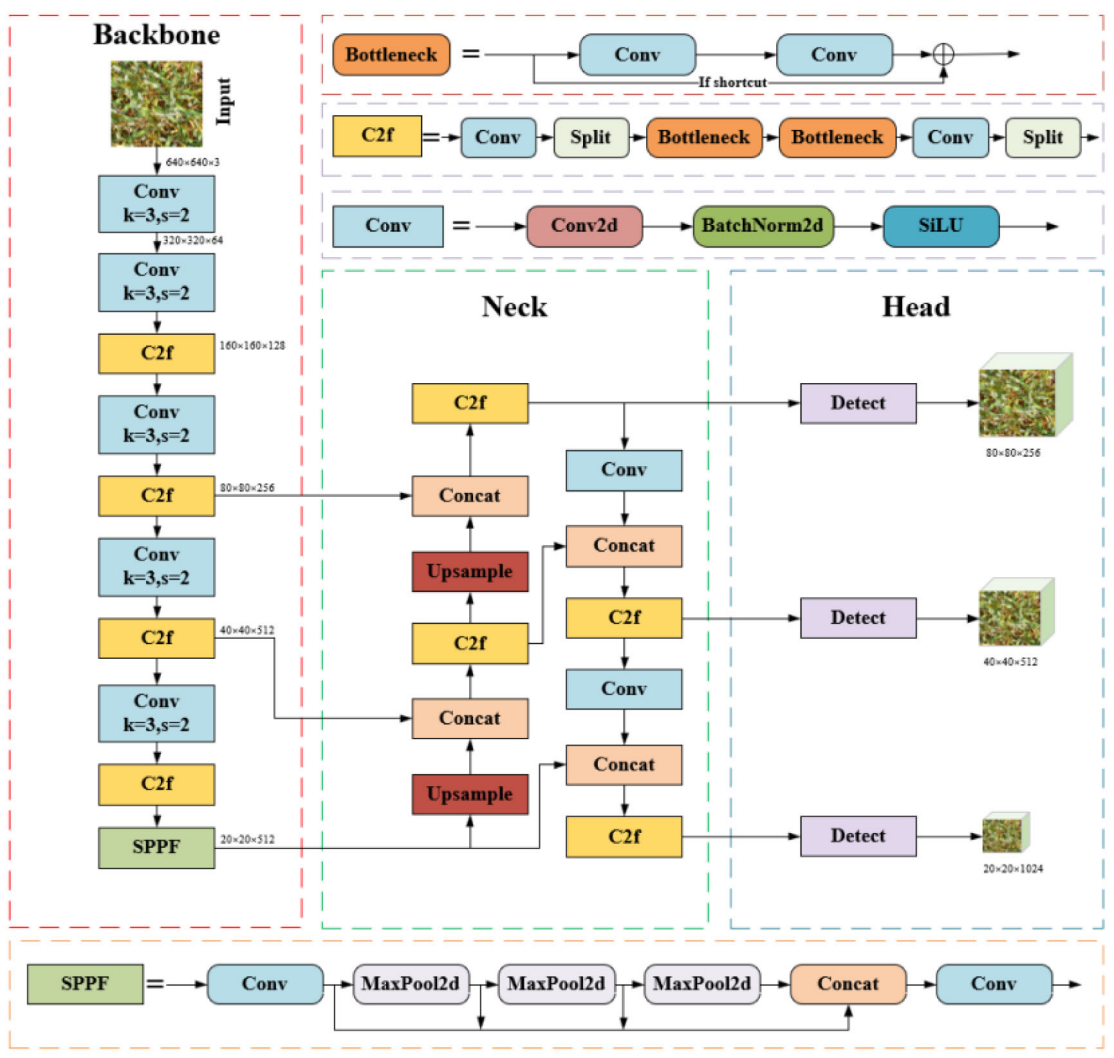
YOLOv8 network structure diagram.

The YOLOv8 model is built upon earlier YOLO series (Yaseen, 2024), incorporating improvements through optimizations in network architecture and training strategies, which further enhance object detection performance and efficiency. The YOLOv8 network architecture is made up of three primary components: Backbone, Neck, and Head. Backbone extracts high-level semantic features from the image, including object shapes, textures, and contextual information. The neck fuses features from various layers for multiscale processing, enhancing the ability to detect targets at different scales. The head uses the fused features to predict bounding boxes and classes, generating the final detection results. Spatial Pyramid Pooling Force (SPPF) generates fixed feature representations for multi-scale objects without the need to resize images or introduce spatial information loss, thereby accelerating computational speed. The C2f block combines high-level features with contextual information to improve detection accuracy. The convolutional blocks consist of 2D convolution layers, 2D batch normalization, and activation functions. The detection algorithm employs an Anchor-Free method, getting rid of the dependence on conventional Anchor Boxes. This improvement effectively addresses the localization errors and the imbalance between positive and negative sample distributions inherent in anchor-based methods. The YOLOv8 series offers five versions: n, s, m, l, x, based on different network depths and feature map widths. Among these, YOLOv8n has the fewest parameters and of the highest detection speed. Therefore, to balance the model’s parameter count while enabling rapid data processing, this study selected the lightweight YOLOv8n version. The YOLOv8 network architecture is illustrated in **Figure 2**.

#### 2.2.2 Improved YOLOv8 network architecture

**Figure 3:**
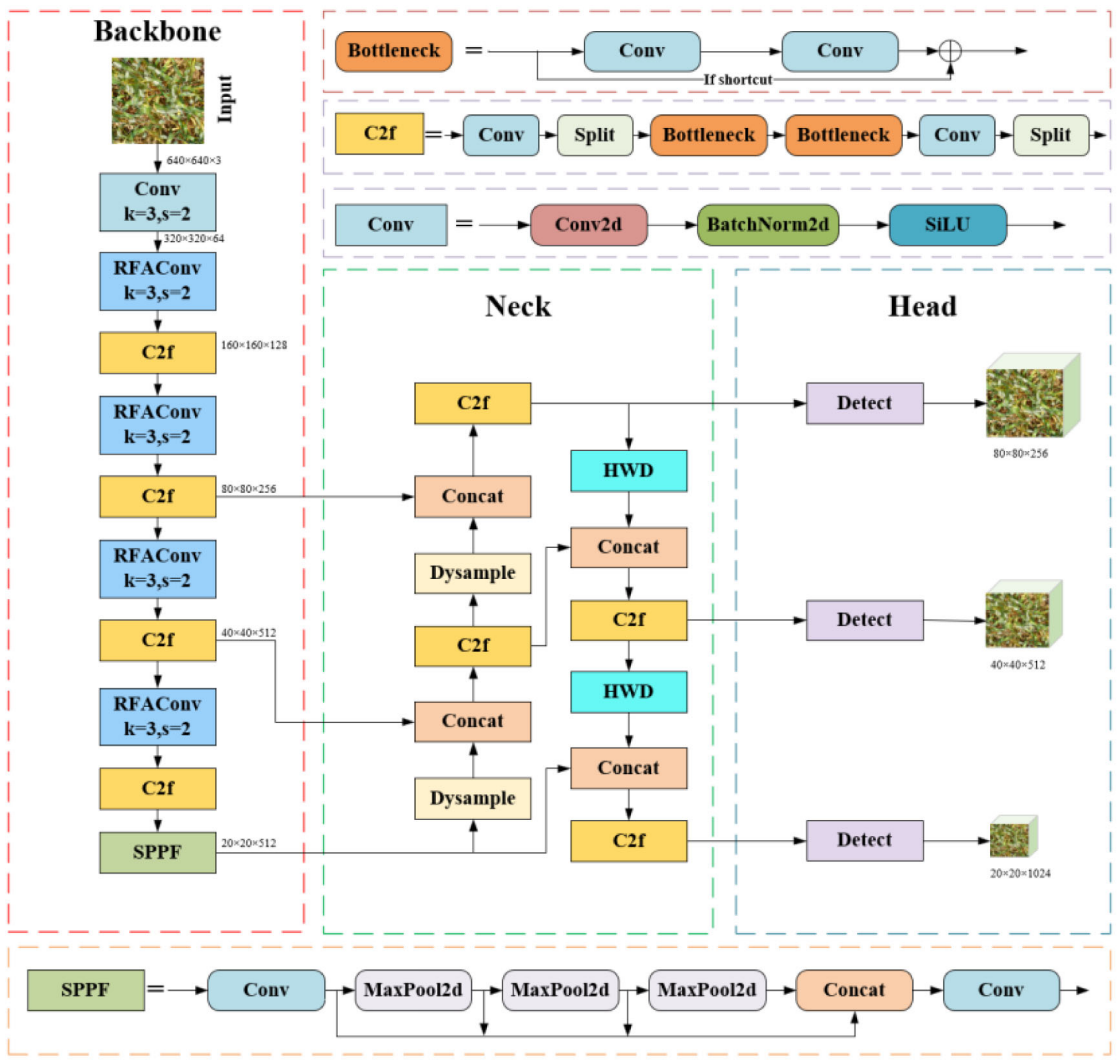
Structure of YOLOv8-FDA.

This study aims to enhance the original YOLOv8 in four aspects. First, in the Backbone, the original Conv module after the first layer is replaced with RFAConv to improve feature extraction capabilities. Next, in the Neck, the original Upsample module is replaced with Dysample, which dynamically adjusts the scale of the feature maps, better preserves detail information, improves the model’s adaptability to objects of multiple scales, and accelerates the convergence speed. Additionally, the original downsampling Conv module is replaced with HWD, which incorporates Haar wavelet transformation for more effective downsampling, reducing parameters while preserving frequency-domain features and detailed information. Finally, the SDL function replaces the default YOLOv8 loss, jointly optimizing bounding box distance and shape fitting, which significantly enhances localization accuracy and accelerates model convergence. The improved model in this study is named YOLOv8-FDA, as illustrated in **Figure 3**.

#### 2.2.3 Receptive Field Attentive-RFAConv

**Figure 4:**
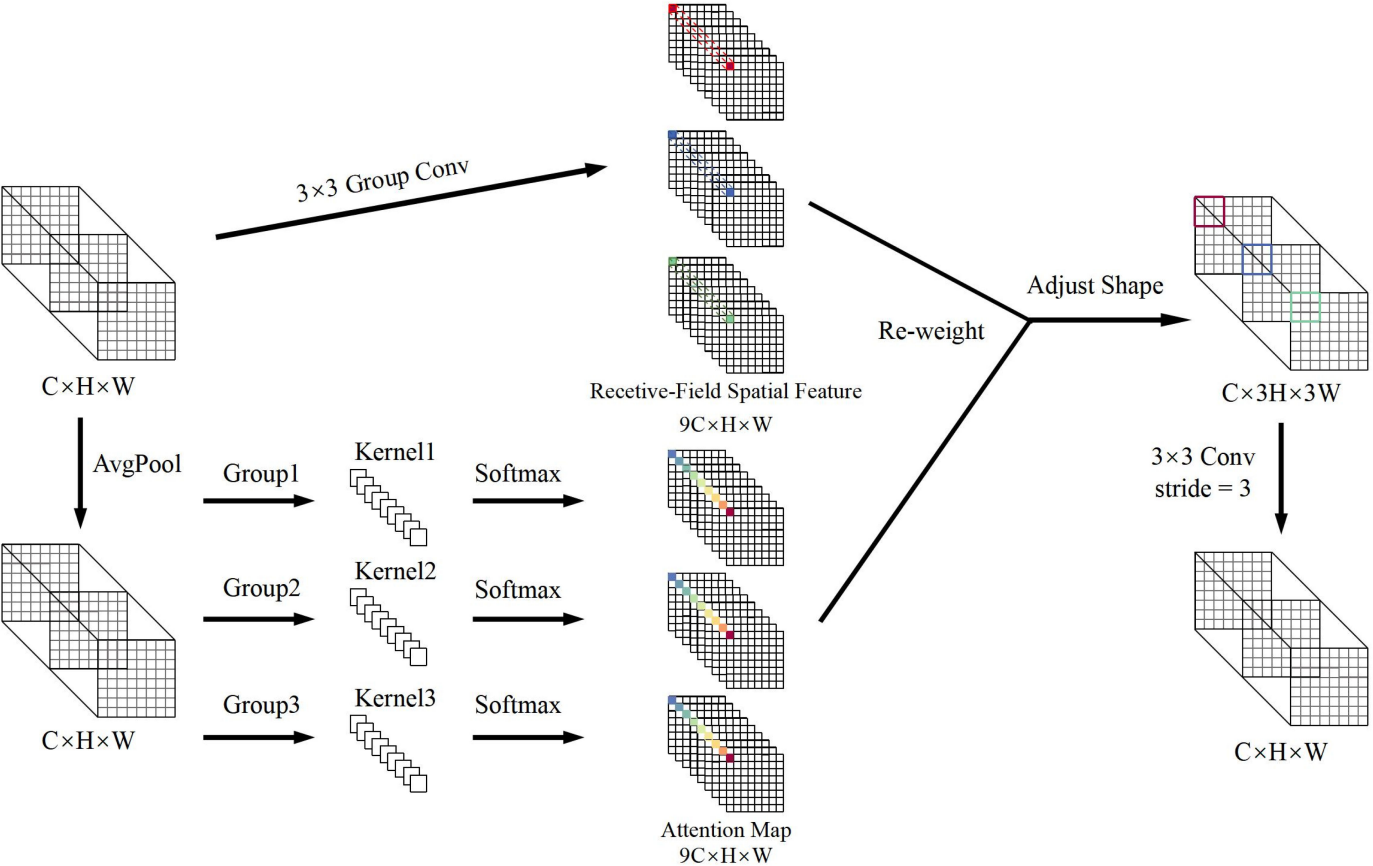
RFAConv structure.

Field detection of wheat spikes faces unique challenges, including diverse spike morphologies and intricate details in dense scenes. Traditional convolutional networks have limitations due to their parameter sharing mechanism, struggle to adapt to the geometric deformations and scale variations of wheat spikes, leading to insufficient capability in capturing fine-grained spike features (Khan et al., 2020). Moreover, the uniform application of fixed convolutional kernels across spatial locations fails to adequately model the local feature differences, further weakening the model’s robustness in complex agricultural environments. To address these problems, this study presents the RFAConv module (Zhang et al., 2023), which significantly enhances the accuracy of wheat spike detection through adaptive receptive field optimization and attention-weighted mechanisms. The structural design is shown in **Figure 4**.

The key innovation of RFAConv resides in the combination of spatial attention mechanisms and traditional convolution operations, which remarkably boosts the network’s capacity to perceive local features and elevates the accuracy of representation. This module can dynamically recognize the prominent regions within the input feature map and adaptively modify the weight distribution of the convolution kernels, enabling a focused enhancement of key features. This design balances the need for a large receptive field with efficient computational resource allocation, thereby improving model performance while maintaining low computational overhead.

**Figure 5:**
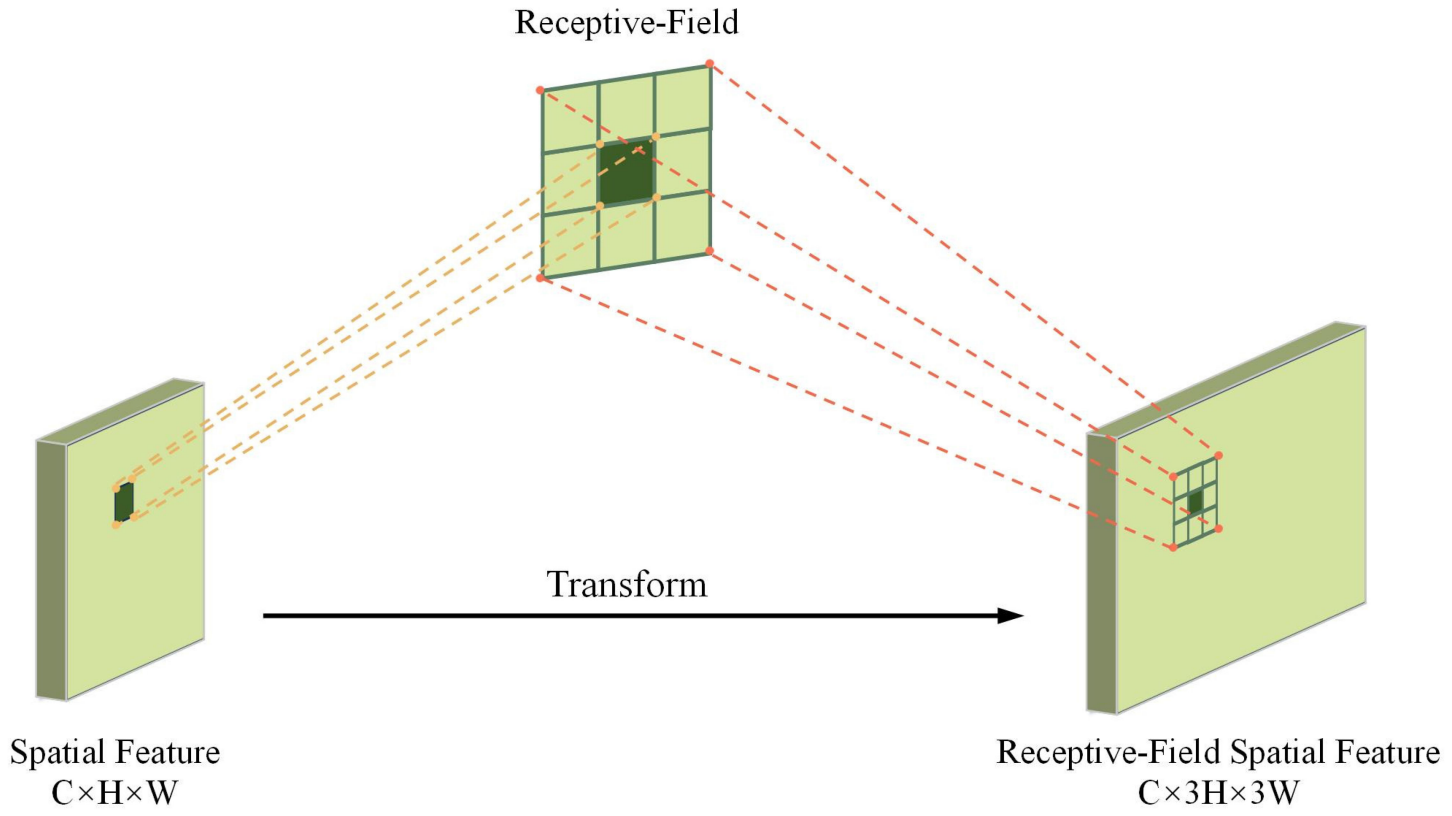
Receptive field spatial feature transformation.

As shown in **Figure 5**, the spatial characteristics of the receptive field are composed of sliding windows that do not overlap 3 × 3, each window being responsible for extracting detailed information from local regions (Bertels et al., 2022). By introducing group convolution techniques, the module expands the feature set that matches the receptive field size and efficiently compresses the feature dimensions using fast group convolution algorithms. This process transforms the original features into a new representation enriched with spatial attention, providing more discriminative input for downstream tasks.

To improve feature detect accuracy, average pooling is used to aggregate global contextual information within the receptive field, significantly reducing computational complexity and the number of parameters. Then, 1 × 1 group convolutions are applied to enable cross-channel feature interaction, and the Softmax function is used to adaptively weight the feature importance within the receptive field, thereby enhancing the response strength of key regions. The calculation of RFA, as shown in **Equation 1**, is formulated as follows:


(1) $$F=\text{softmax }\left(g^{1\times 1}\left(\text{AvgPool }(X\right))\right)\times \text{ReLU }\left(\text{Norm }\left(g^{k\times k}\left(X\right)\right)\right)=A_{rf}\times F_{rf}$$

In this case, $g^{1\times 1}$ represents a group convolution with a size of 1 × 1, *Norm* refers to normalization, *k* denotes the size of the convolution kernel and X represents the input feature map. The feature map *F* is obtained by multiplying the attention map *A_rf_* with the spatial features *F_rf_* that have been transformed by the sensory fields. The final receptive field spatial feature map has dimensions of *C* × 3*H* × 3*W*, where the width and height are three times the size of the input feature map. To adjust the dimensions of the feature map, a 3 × 3 convolution is applied. Through the attention map learned by RFAConv, the model combines the feature information from each receptive field region, significantly improving the accuracy of feature extraction.

#### 2.2.4 Dynamic upsampling-DySample

**Figure 6:**
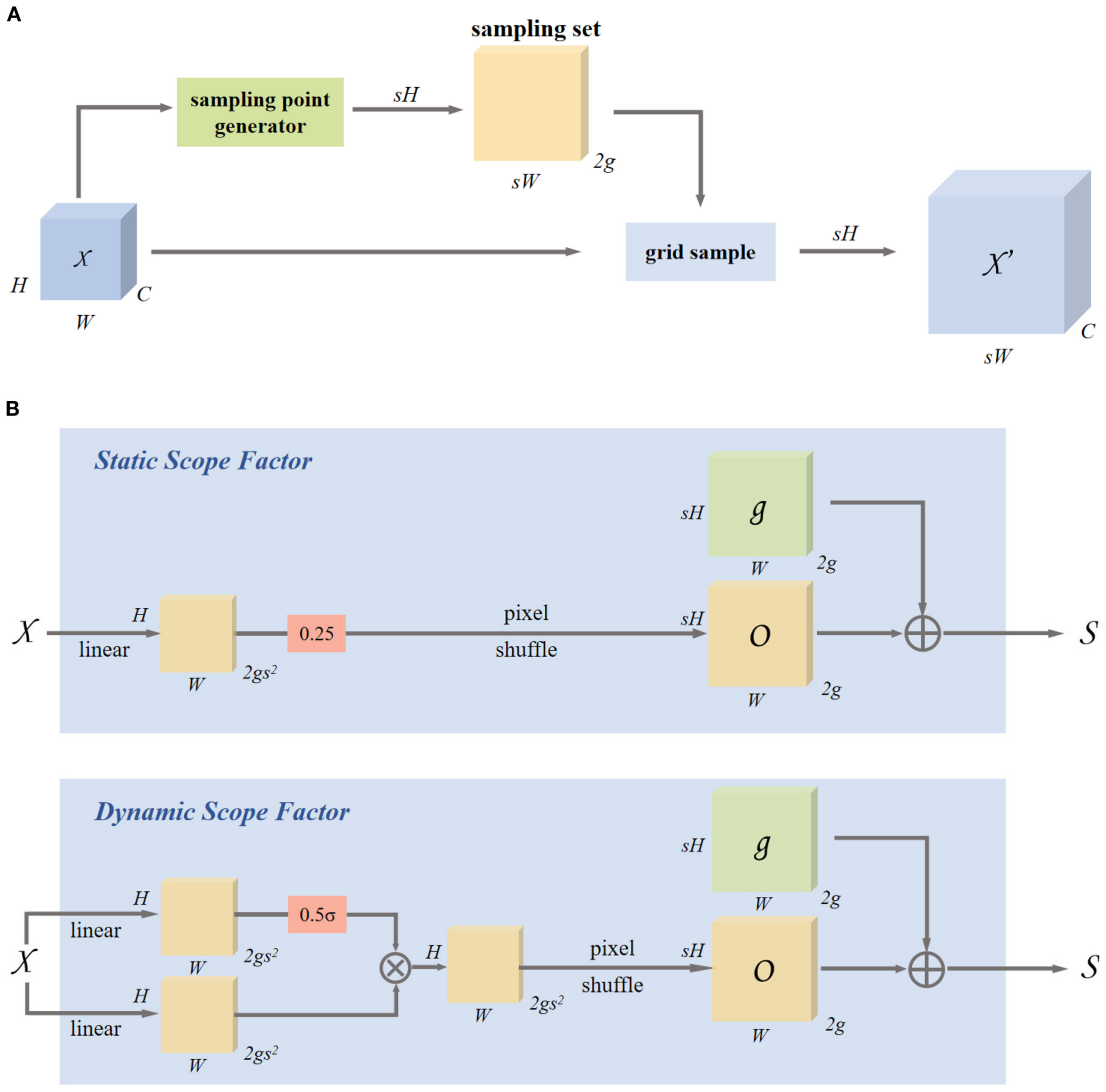
Dynamic upsampling based on sampling and module design in DySample. **(A)** Sampling based dynamic upsampling. **(B)** Sampling based dynamic upsampling.

This research employs the DySample (Liu et al., 2023) dynamic upsampling technique for tackling the computational redundancy, poor multi-scale adaptability, and insufficient detail preservation in traditional upsampling methods for wheat spike detection. In the YOLOv8-FDA, DySample substitutes for the standard Upsampling module, overcoming the high resource demands of traditional methods and significantly enhancing the practicality of deploying the model on mobile devices in agricultural fields. This improvement is particularly crucial for dense wheat spike scenarios, where traditional methods often lead to edge blurring and texture loss when processing small-scale spike grains, hindering the extraction of key morphological features. DySample reconstructs the upsampling process through a feature-driven adaptive sampling mechanism, eliminating the need for complex convolution operations and significantly reducing parameter size and computational overhead. Its core innovation lies in dynamically adjusting the sampling locations based on the spatial arrangement of wheat spikes, enhancing the representation of details such as spike grain connections and awns while preserving high-frequency information. The lightweight architecture design effectively reduces the dependence on high-resolution input images and significantly optimizes resource utilization for real-time field detection, with detailed design shown in **Figure 6**.

DySample uses a feature map *χ* of size *C* × *H*_1_ × *W*_1_, and a sampling set *S* of size 2 × *H*_2_ × *W*_2_, where the first dimension of size 2 represents the x and *y* coordinates, the grid sampling function uses the coordinates in *S* to re-sample the bilinearly interpolated version of *χ*, resulting in a new feature map of size *C* × *H*_2_ × *W*_2_. This process is described as follows:


(2) $$\chi^{\prime }=\text{grid\_sample}\left(\chi ,S\right)$$

DySample initially maps the input feature map *χ* into a continuous space through bilinear interpolation and computes an offset *O* to modify the sampling locations. This offset *O* is then superimposed on the original sampling grid *G* to produce the final set of sampled points *S*. As shown in **Equation 3**, the formula is as follows:


(3) $$O=0.5\times \text{sigmoid }\left({\text{linear}}_{1}\left(\chi \right)\right)\times {\text{linear}}_{2}(\chi )$$

To enhance the adaptability of the offset, DySample generates a point-wise dynamic range factor within the interval [0, 0.5] using linear projection, which is then adjusted using a *sigmoid* function. As defined in **Equation 4**, the offset *O* is calculated as follows:


(4) S=G+O

Finally, the upsampled feature map $X^{\prime }$ of size *C* × *sH* × *sW* is generated using the sampling set through grid sampling, as shown in Equation 2. DySample adapts to capture key details through dynamic sampling position adjustment, significantly enhancing its ability to represent complex textures of wheat spikes and small-scale targets.

#### 2.2.5 HWD module

**Figure 7:**
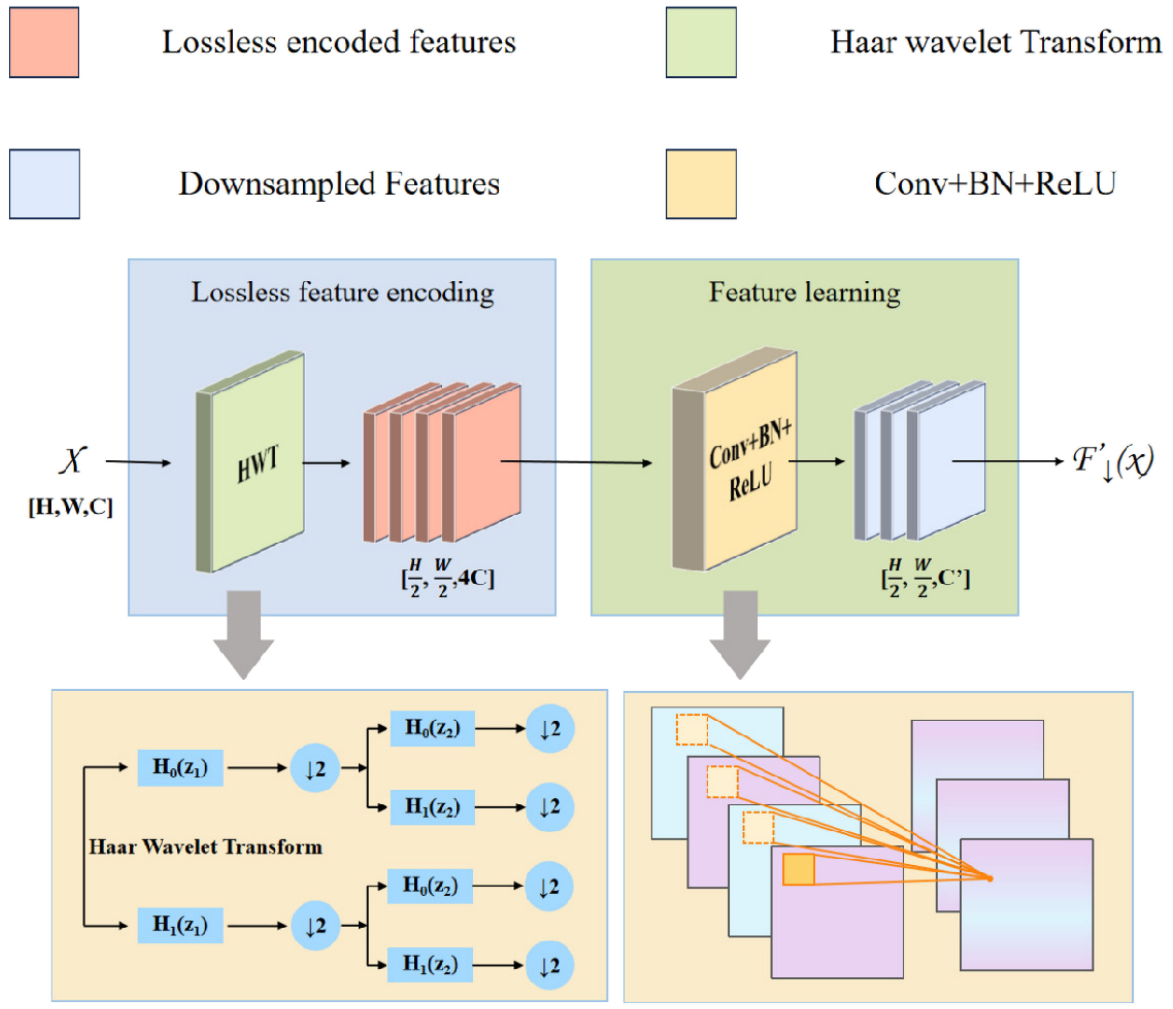
HWD network structure.

In the YOLOv8n architecture, the traditional downsampling with stride-2 convolutions drastically reduces feature map size, leading to the disappearance of intricate and detailed elements and a limited receptive field, which significantly weakens the model’s ability to capture subtle local features. Inspired by the Haar wavelet downsampling (HWD) model (Xu et al., 2023), whose network architecture is shown in **Figure 7**, this study replaces the standard downsampling process with the Haar wavelet transform. This technique performs frequency-domain decomposition, downsampling the feature map while preserving key image details and expanding the model’s perception of global features. Compared to strided convolutions, HWD offers a simpler and more efficient design that reduces computational costs, accelerates training convergence, and enhances detection performance. Based on these advantages, this study replaces the stride-2 convolution layers in the Neck with the HWD module, comprehensively optimizing the model’s detection capabilities in complex scenarios.

**Figure 8:**
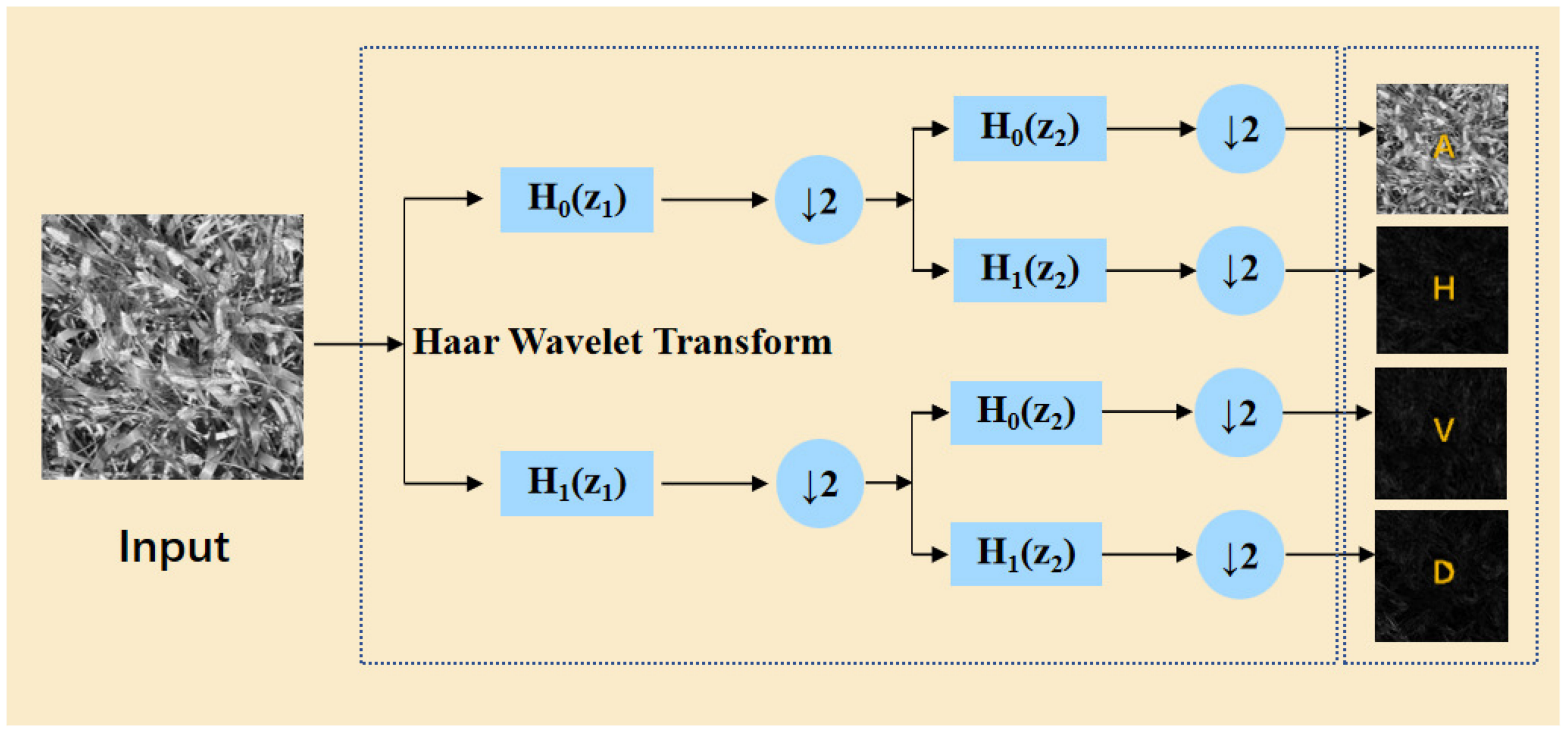
Wavelet transform method diagram.


The essence of the HWD module is composed of a lossless feature encoding area and a feature learning area. The lossless encoding area utilizes the Haar wavelet transform, which, at the target resolution, applies cascaded high-pass (*H*_0_) and low-pass (*H*_1_) filtering followed by downsampling (↓ 2) to generate a low frequency approximation subband (A) and directional high-frequency subbands (horizontal-H, vertical-V, diagonal-D) are generated. This process, illustrated in **Figure 8**, preserves fine-grained features while compressing spatial resolution. As shown in **Equation 5**, the formulation of this process is presented as follows:

(5) $$\begin{array}{c} \{\begin{array}{l} \phi_{1}\left(x\right)=\frac{1}{\sqrt{2}}\phi_{1,0}\left(x\right)+\frac{1}{\sqrt{2}}\phi_{1,1}\left(x\right)\\ \psi_{1}\left(x\right)=\frac{1}{\sqrt{2}}\phi_{1,0}\left(x\right)-\frac{1}{\sqrt{2}}\phi_{1,1}\left(x\right) \end{array}\\ \phi_{j,k}\left(x\right)=\sqrt{2^{j}}\phi \left(2^{j}x-k\right),k=0,1,\ldots ,2^{j}-1. \end{array}$$

where *j* represents the scale level of the wavelet decomposition, and *k* represents the direction selection of the wavelet function.

The feature learning area applies 1 × 1 Conv-BN-ReLU operations. By adaptively adjusting feature channel dimensions, this module efficiently filters redundant information, thus improving the ability of the subsequent layers to extract discriminative features. This architecture significantly boosts model learning efficiency and generalization performance, establishing a robust foundation for accurate data analysis.

Given an input feature map of size *H* ×*W* × *C*, the downsampled output size is $\frac{H}{2}\times \frac{W}{2}\times C$ . Compared to traditional downsampling using a stride-2 3 × 3 convolution, the optimization of total parameters and FLOPs overhead in HWD is shown in **Table 1**.

**Table 1:** Comparison of parameters and FLOPs for two downsampling methods.

Module	Parameters	FLOPs
HWD	4*C* ^2^	2*HWC* ^2^ + 3.75*HWC*
Stride-2 3 × 3 Convolution	9*C* ^2^	4.5*HWC* ^2^ − 0.25*HWC*

**Table 1** shows that the quantity of parameters in strided convolution is more than twice that of the HWD module. When the quantity of channels *C >* 1, the computational overhead is also significantly higher than that of the HWD module. In conclusion, HWD module achieves a better trade-off between parameter count and computational complexity while outputting multi-band feature representation.

#### 2.2.6 Scale-based Dynamic Loss

**Figure 9:**
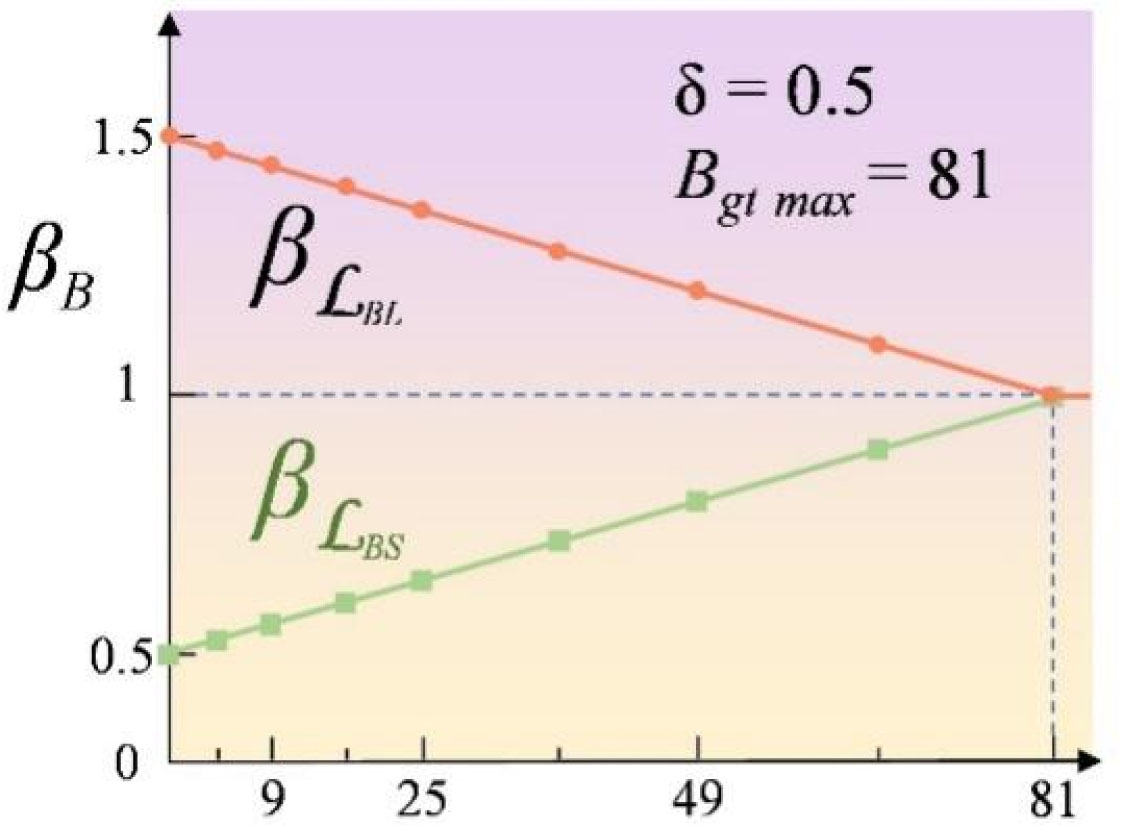
The value of weight in Sloss and Lloss concerning the target BBox area.


The initial YOLOv8 utilizes the Complete Intersection over Union (CIoU) loss for bbox regression (Zheng et al., 2021), jointly optimizing the Intersection over Union (IoU), the Euclidean distance between the centroids of the bounding boxes, and the aspect ratio similarity (Wang and Song, 2021). However, it has a limitation regarding scale sensitivity: small targets experience significant IoU fluctuations due to label inaccuracies, leading to decreased regression stability. To tackle this problem, this study presents the Scale-based Dynamic Loss (SDL) (Yang et al., 2025) which dynamically adjusts the loss component weights according to target size. As shown in **Figure 9**, as the target size decreases, the weight of the scale loss (*β*ℒ*_BS_*) decreases to suppress large fluctuations, while the weight of the location loss (*β*ℒ*_BL_*) increases to enhance location stability. This adaptive mechanism improves detection robustness for small targets and mitigates training instability caused by label noise. As shown in **Equations 6**–**8**, the formulas before and after the improvement are as follows:

(6) $${ℒ}_{CIoU}=1-IoU+\frac{\rho^{2}\left(b_{p},b^{gt}\right)}{c^{2}}+\alpha \text{v}$$

(7) $${ℒ}_{BS}=1-IoU+\alpha v$$

(8) $${ℒ}_{BL}=\frac{\rho^{2}\left(b_{p},b^{gt}\right)}{c^{2}}$$

Here, *IOU* is the ratio of the area where the predicted bbox and ground truth bbox overlap to the total area covered by both boxes combined, *αv* is the aspect ratio penalty term, which improves the morphological consistency through the angular disparity metric with adaptive weights, $\rho^{2}\left(b_{p},b^{gt}\right)$ represents the Euclidean distance between the center of the predicted bbox and the center of the ground truth bbox, while *c* denotes the diagonal length of the minimum enclosing rectangle that contains both the predicted and ground truth boxes.

The size of the target changes when the model scales the image or subsamples the feature map. As shown in **Equations 9**, **10**, the ratio between the original image and the current feature map and the influence coefficient of BBox ( $\beta_{B}$ ) are calculated as follows:


(9) $$R_{OC}=\frac{w_{o}\times h_{o}}{w_{c}\times h_{c}}$$

(10) $$\beta_{B}=\text{min }\left(\frac{B_{gt}}{B_{gtmax\;}}\times R_{OC}\times \delta ,\delta \right)\;$$

where *w_o_*, *w_h_* are the width and height of the original image, and *w_c_*, *w_h_* are the width and height of the current feature map. The influence coefficient of the loss is based on the area of the current bbox, with its range constrained to *δ*, which is adjustable. As shown in **Equations 11**–**13**, the final scale-based dynamic loss for the bbox is given by the following formula:


(11) $$\beta_{{ℒ}_{BS}}=1-\delta +\beta_{B}$$

(12) $$\beta_{{ℒ}_{BL}}=1+\delta -\beta_{B}$$

(13) $${ℒ}_{SDB}=\beta_{{ℒ}_{BS}}\times {ℒ}_{BS}+\beta_{{ℒ}_{BL}}\times {ℒ}_{BL}$$

In this way, SDL dynamically adjusts the ratio of scale loss and location loss based on the size of the target, effectively improving the regression accuracy for small targets such as wheat spikes.

## 3 Experiments and results

### 3.1 Experimental configuration

The experimental hardware and software environment and the model training parameters are shown in **Tables 2**, **3** respectively.

**Table 2:** Model training software and hardware environment configuration.

Software and hardware environment	Configuration
Operating system	Windows11
Software	Python3.10.0, PyTorch2.6.0, CUDA12.1
CPU	Intel(R) Core™ i7-14700KF @ 3.40GHz
GPU	NVIDIA GeForce RTX 4070 SUPER 12GB
RAM	32GB

**Table 3:** Training parameters.

Model training parameters	Configuration
Parameter learning rate	0.01
Input image size	640 × 640
epoch	150
Momentum	0.937
batch size	16
Warmup bias lr	0.1
Weight decay	0.0005

The training parameters listed in **Table 3** were primarily based on the default recommendations of YOLOv8 (Yaseen, 2024). To ensure stable convergence on the GWHD dataset, several key hyperparameters, such as the learning rate and batch size, were empirically adjusted through preliminary experiments. This parameter selection strategy is consistent with previous practices in similar object detection studies (Li et al., 2024b).

**Figure 10:**
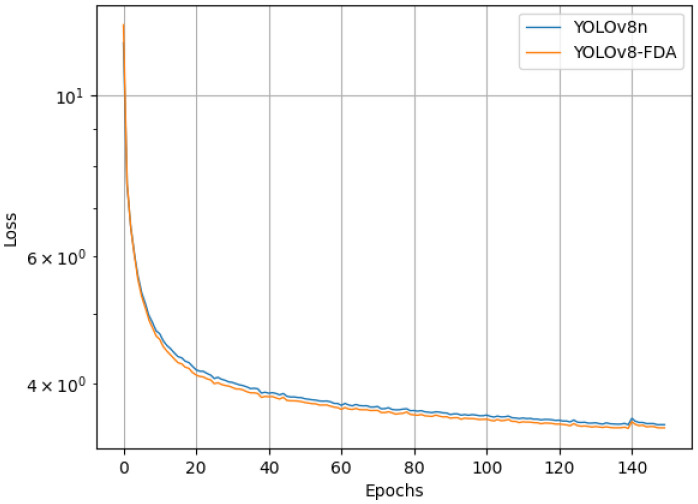
The training loss curves of the baseline model and the YOLOv8-FDA.

The baseline model and the YOLOv8-FDA were trained under the same experimental environment and parameter settings, and their loss variations during the training process are presented in **Figure 10**.

As shown in **Figure 10**, the larger learning rate in the early stages of training causes the loss of both models to decrease rapidly, but also leads to instability. As training progresses, the models gradually approach saturation, the learning rate decreases, and the rate of loss reduction slows down. Eventually, the models converge to a stable state. Compared to the baseline model, the YOLOv8-FDA demonstrates faster convergence and lower loss throughout the training process.

### 3.2 Performance measures

The experiment evaluates miss and false detection rates by comparing YOLOv8-FDA with other models under identical conditions. The evaluation primarily uses three metrics: precision, recall, and mean Average Precision calculated at a single IoU threshold of 0.5 (mAP@0.5). Precision reflects the accuracy of the model, recall reflects the sensitivity of the model, mAP reflects the average detection precision across all categories, and mAP@0.5 denotes the mean average precision when the IoU threshold is set at 0.5. As shown in **Equations 14**–**17**, the mathematical expressions for these evaluation metrics are as follows:


(14) $$P=\frac{TP}{TP+FP}$$

(15) $$R=\frac{TP}{TP+FN}$$

(16) $$AP=\int_{0}^{1}P\left(R\right)dR$$

(17) $$mAP=\frac{1}{m}\sum_{i=1}^{m}AP\left(i\right)$$

In the formula, *TP* denotes the number of correctly predicted foreign object positive instances, which are indeed positive in the outcomes of the detection. *FP* represents the number of instances incorrectly predicted as foreign object positives but actually belonging to non-foreign object classes. *FN* represents the number of instances predicted as foreign object positives but detected as non-foreign object negatives.

Additionally, this paper uses the number of parameters as the evaluation metric for the algorithm’s space complexity and the floating point operations (FLOPs) as the evaluation metric for the algorithm’s computational complexity.

### 3.3 Experimental validation of model components

To validate the enhancement impact of every improvement in the YOLOv8-FDA, we contrast different evaluation metrics of every enhanced model on the test set. In the experiment, each improvement is regarded as a variable to guarantee the consistency of the experimental environment and parameter settings. The outcomes of the experiment are presented in **Table 4**, where “✓” represents the corresponding improvement. **Table 4** shows:

**Table 4:** Ablation experiment results.

Baseline	RFAConv	Dysample	HWD	SDL	Precision	Recall	mAP@0.5	Parameters	FLOPs
Yolov8n	–	–	–	–	84.9%	74.2%	82.3%	3.02M	8.2G
Yolov8n	✓	–	–	–	86%	76.9%	84.4%	3.05M	8.4G
Yolov8n	–	✓	–	–	84.8%	75.4%	83.1%	3.03M	8.3G
Yolov8n	–	–	✓	–	83.8%	74.7%	82.2%	2.91M	8.1G
Yolov8n	–	–	–	✓	84.1%	75%	82.5%	3.02M	8.2G
Yolov8n	✓	✓	–	–	85%	76.8%	83.5%	3.06M	8.5G
Yolov8n	✓	–	✓	–	85.6%	77.1%	84.1%	2.94M	8.3G
Yolov8n	✓	–	–	✓	85.2%	76.4%	82.9%	3.05M	8.4G
Yolov8n	–	✓	✓	–	85.1%	75.5%	82.3%	2.93M	8.1G
Yolov8n	–	✓	–	✓	84.5%	75.3%	82.5%	3.03M	8.3G
Yolov8n	–	–	✓	✓	83.3%	74.7%	81.3%	2.92M	8.1G
Yolov8n	✓	✓	✓	–	85.9%	77.3%	84.1%	2.96M	8.3G
Yolov8n	✓	✓	–	✓	84.3%	76.2%	84.2%	3.06M	8.5G
Yolov8n	✓	–	✓	✓	85.5%	76.9%	83.7%	2.94M	8.3G
Yolov8n	–	✓	✓	✓	83.9%	75.3%	82.5%	2.93M	8.1G
Yolov8n	✓	✓	✓	✓	86.3%	77.5%	84.9%	2.96M	8.3G

**Table 4** shows the results of the ablation experiments, which evaluate the contribution of each individual module and their combinations to the performance of the model. The incorporation of the RFAConv module, which enhances feature representation through a receptive field attention mechanism, leads to significant performance gains. Specifically, Precision improves by 1.1%, Recall increases by 2.7%, and mAP@0.5 rises by 2.1%. This improvement comes at the cost of an increase of 0.2G in model FLOPs, indicating that RFAConv helps the model better adapt to the morphological diversity of wheat spikes, enhancing the detection accuracy for wheat ears.

The DySample module, which optimizes the feature map resolution adjustment process, results in a 1.2% increase in Recall while maintaining nearly the same number of parameters and FLOPs. This shows that DySample plays a crucial role in improving the model’s ability to detect small-scale wheat spikes without adding significant computational overhead. Meanwhile, the HWD module, which performs dimensionality reduction using Haar wavelet transform, reduces model parameters by 0.11M and FLOPs by 0.1G, while still retaining 82.2% of the baseline mAP@0.5 performance, demonstrating its efficiency in reducing model complexity without sacrificing detection accuracy. Finally, the SDL module optimizes the IoU calculation for bounding boxes, significantly improving localization accuracy in dense wheat spike scenes. This results in a 0.8% increase in Recall and a 0.2% increase in mAP@0.5, enhancing the model’s ability to distinguish overlapping wheat spikes.

In addition to evaluating the performance of individual modules, we also considered combinations of modules to assess their synergistic effects. The combination of RFAConv and DySample, for example, resulted in a 2.2% increase in Recall, showing that these modules work well together to enhance the model’s sensitivity to small targets. Furthermore, the combination of RFAConv, DySample, and SDL demonstrated the greatest improvements, with both Recall and mAP@0.5 increasing significantly. These results highlight the importance of combining complementary modules for maximizing detection accuracy, particularly in complex wheat spike scenes with overlapping spikes. Overall, the combination of these modules leads to consistent improvements in both detection accuracy and computational efficiency, with the final model achieving 86.3% Precision, 77.5% Recall, and 84.9% mAP@0.5, as shown in the last row of **Table 4**.

**Figure 11:**
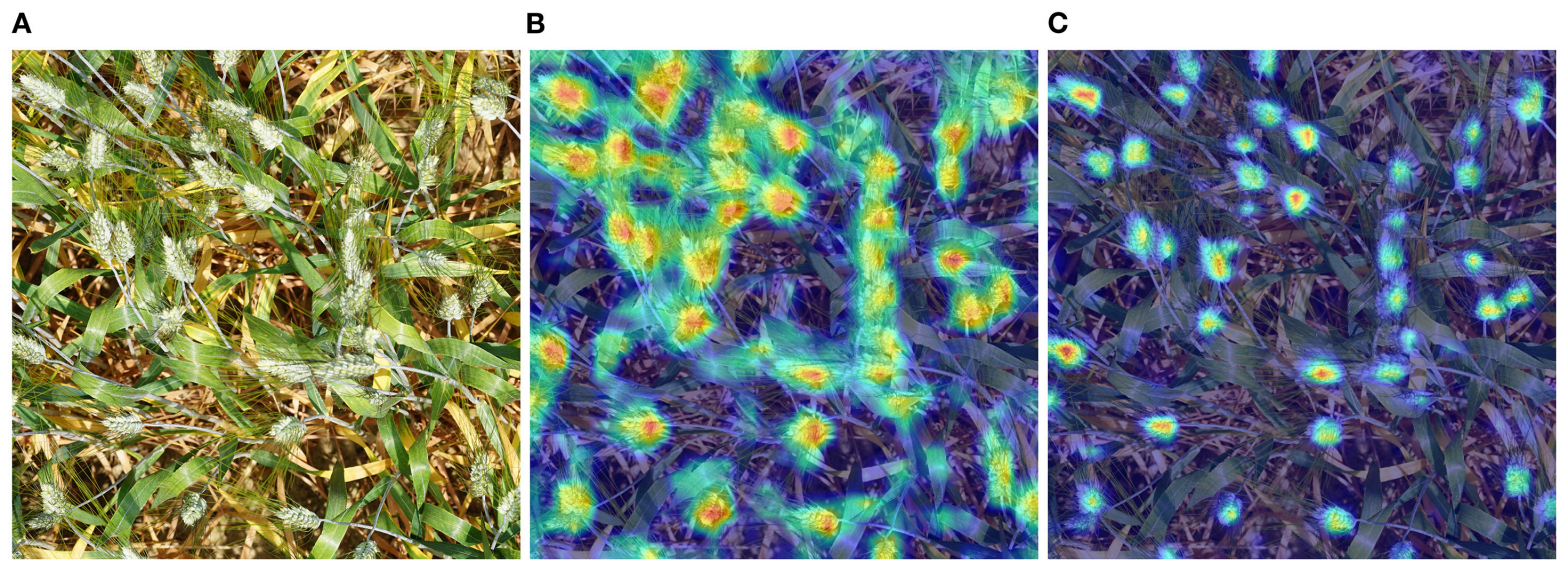
Visualization of model prediction results based on XGradCAM algorithm. **(A)** Input image. **(B)** YOLOv8n Visualization Results. **(C)** YOLOv8-FDA Visualization Results.

The YOLOv8-FDA achieves a breakthrough in complex agricultural environments: while reducing model parameters by 0.06M, Precision increased by 1.4%, Recall improved by 3.3%, and mAP@0.5 rose by 2.6%. This compact yet efficient structure significantly boosts the generalization ability of the model, improving its applicability and reliability in practical applications, providing a robust and practical detection solution for precision agriculture. To understand the internal workings of the model, the predicted results of the original model and the proposed model in this paper are visualized using the XGradCAM (Fu et al., 2020) algorithm, as shown in **Figure 11**.

As illustrated in **Figure 11**, the YOLOv8-FDA effectively suppresses interference from background and redundant information, directing core attention to the spike axis of the wheat ear. In contrast, the original model performs suboptimally due to excessive focus on non-critical regions such as awns, stems, and leaves. This shows that YOLOv8-FDA has the ability to capture the distinctive features of wheat ears with greater precision, and its visualization results align closely with experimental expectations.

### 3.4 Comparative analysis of model performance

**Figure 12:**
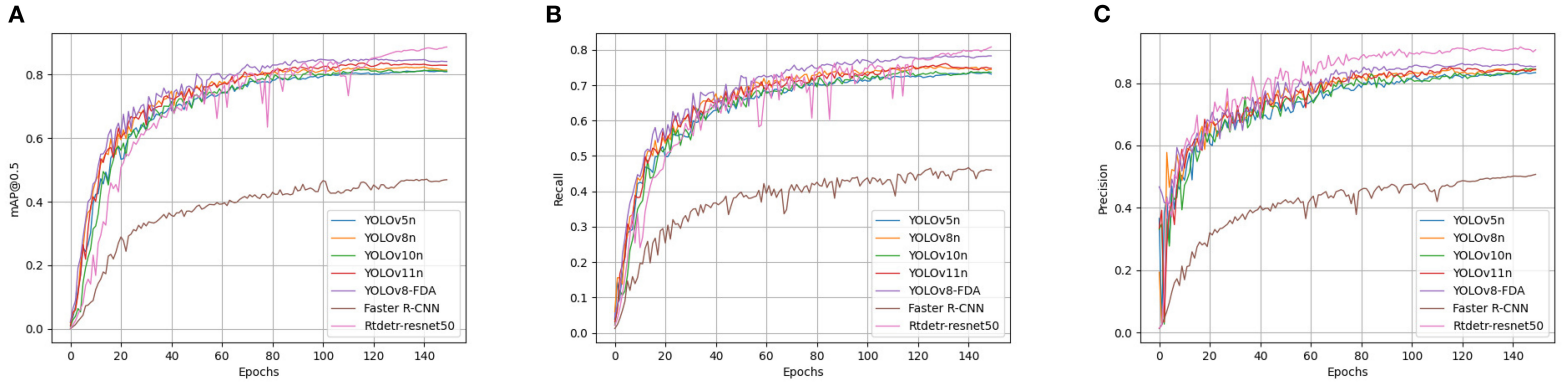
Comparison among the evaluation indices of diverse detection models. **(A)** Precision rate curves for each model. **(B)** Precision rate curves for each model. **(C)** mAP@0.5 curves for each model.

Under the experimental setting of 150 training epochs, the core performance metrics of various detection models on the validation set all reached a stable state at the end of training, as shown in **Figure 12**. This indicates that all models effectively converged on the dataset, ensuring reliable performance comparison. For the baseline selection, YOLOv5, YOLOv8, YOLOv10, and YOLOv11 were chosen because they follow a clear architectural lineage from the Ultralytics framework (Kang and Kim, 2023), are widely adopted in the community, and have stable open-source implementations. In contrast, YOLOv6 (Li et al., 2022a), YOLOv7 (Wang et al., 2023a), and YOLOv9 (Wang et al., 2024) were excluded due to limited community adoption, lack of long-term maintenance, or divergence from the main codebase, which would reduce the reproducibility and relevance of comparisons.

To verify the effectiveness of YOLOv8-FDA, it was tested and compared with models such as YOLOv5n, YOLOv8n, YOLOv10n, YOLOv11n, Faster R-CNN, and Rtdetr-resnet50. The results are shown in **Table 5**. YOLOv8-FDA achieves 86.3% Precision, 77.5% Recall, and 84.9% mAP@0.5, outperforming lightweight models such as YOLOv5n, YOLOv8n, YOLOv10n, and YOLOv11n in both accuracy and recall. Although YOLOv11 demonstrates stronger performance than YOLOv5, YOLOv8, and YOLOv10, and achieves this with fewer parameters and FLOPs, our goal is to enhance the performance of lightweight baselines that are widely deployed in practice. Therefore, YOLOv8n was selected as the core baseline for improvement, as it represents a balance of simplicity, efficiency, and relevance for lightweight enhancement (Lu et al., 2023).

**Table 5:** Results of comparison experiments.

Model	Precision (%)	Recall (%)	mAP@0.5 (%)	Parameters (M)	FLOPs (G)
YOLOv5n	82.7	73.8	81.2	2.51	7.1
YOLOv8n	84.9	74.2	82.3	3.02	8.2
YOLOv10n	82.3	73.7	81.6	2.71	8.3
YOLOv11n	84.7	75.4	83.7	2.59	6.4
Faster R-CNN	50.7	46.7	47.1	41.48	202
Rtdetr-resnet50	91.6	80.8	88.7	42.9	130.7
YOLOv8-FDA	86.3	77.5	84.9	2.96	8.3

Compared with Faster R-CNN, which achieves only 50.7% Precision, 46.7% Recall, and 47.1% mAP@0.5 despite its large model size (41.48M parameters) and high computational cost (202 GFLOPs), YOLOv8-FDA demonstrates clear superiority in both detection accuracy and efficiency. The relatively poor performance of Faster R-CNN may be attributed to the mismatch between its two-stage detection framework and the dense small-object characteristics of the GWHD 2021 dataset. Furthermore, its large computational overhead requires longer training and careful hyperparameter tuning to achieve convergence, and insufficient training epochs or suboptimal optimization may further reduce its accuracy and recall. Although Rtdetrresnet50 achieves the highest Precision (91.6%) and Recall (80.8%), its computational burden is heavy, with 42.9M parameters and 130.7 GFLOPs, making it unsuitable for lightweight and efficient deployment. In contrast, YOLOv8-FDA maintains competitive accuracy with only 2.96M parameters and 8.3 GFLOPs, striking an excellent balance between accuracy and efficiency. In conclusion, YOLOv8-FDA not only enhances counting accuracy but also sustains high detection efficiency, making it well-suited for wheat spike detection and counting tasks under practical conditions.

### 3.5 Robustness test

**Figure 13:**
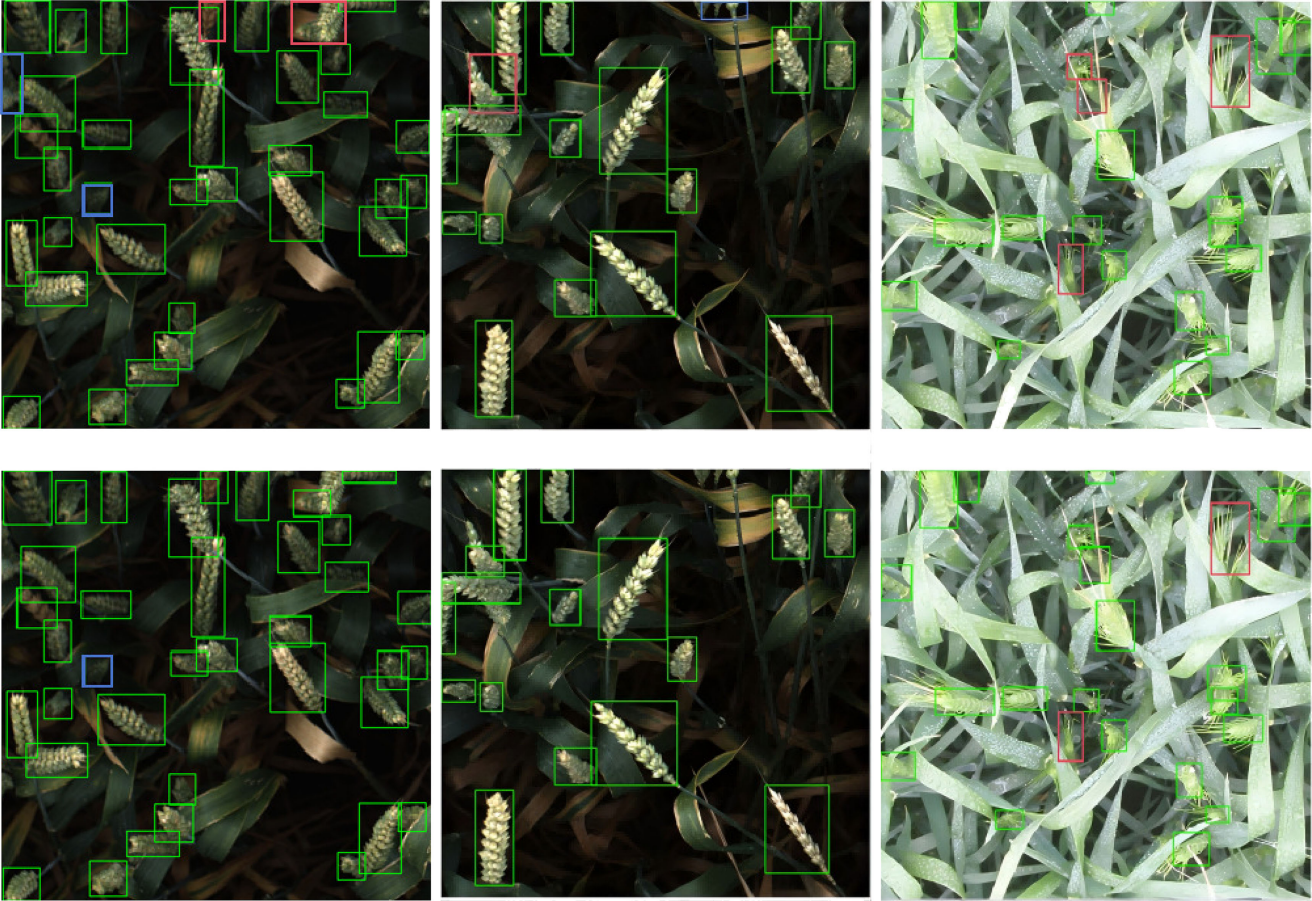
Detection examples of baseline and improved models in complex scenes.

To evaluate the model’s robustness against interference, robustness tests were conducted in complex scenarios such as overlap and occlusion. A total of 637 images from the validation set were used, with both the baseline model and the YOLOv8-FDA applied for detection. The results were statistically analyzed and are presented in **Table 6**, with corresponding visual examples shown in **Figure 13**. In the visual representations, the green boxes stand for accurate detections, whereas the red and blue boxes denote missed detections and false detections, respectively.

**Table 6:** Robustness test results.

Model	Total	TP	FP	FN	Accuracy (%)
YOLOv8	27076	23724	5228	3352	87.6
YOLOv8-FDA	27076	23920	4878	3156	88.3

As shown in **Table 6**, compared to the YOLOv8, the YOLOv8-FDA demonstrates an improvement of 0.7% in correct detection rate. The improvement is illustrated more intuitively through the example in **Figure 13**, where the enhanced model remarkably decreases both the miss detection rate and the false positive rate compared to the YOLOv8. These results illustrate that the YOLOv8-FDA shows a significant enhancement in handling complex field scenarios, exhibiting strong robustness.

## 4 Discussion

To address the issue of detection accuracy degradation caused by the dense arrangement of small-scale wheat ears and occlusion in complex field environments, this paper proposes the YOLOv8-FDA model. The model optimizes multi-scale feature fusion through dynamic upsampling techniques, significantly reducing the edge blurring problem caused by traditional methods, thus ensuring accurate localization of dense wheat ears. The Haar wavelet-based downsampling strategy reduces the number of parameters while preserving frequency-domain features, compressing the model size to 2.96M with a computational cost of only 8.3GFLOPs, rendering it appropriate for deployment on mobile devices. By dynamically adjusting the loss weights, the model effectively mitigates the regression instability caused by annotation errors in small targets. This improvement has universal significance for object detection with significant size differences in agricultural scenarios. In addition, we compared YOLOv8-FDA with several recent methods for wheat ear detection and counting. Multi-scale Feature Enhancement Network (Qian et al., 2024) employs a deformable spatial attention mechanism and multi-scale feature fusion to improve accuracy under occlusion, yet its 37.65M hinder practical deployment on resource-limited hardware. By contrast, YOLOv8-FDA achieves comparable precision while dramatically reducing model size. Li et al. (2024a) proposed a morphology-based approach that yields high spike-count predictions but proves sensitive to ear overlap and lighting variations. YOLOv8-FDA effectively alleviates detection failures caused by overlapping spikes, although its robustness to variable illumination remains constrained; future work may incorporate image preprocessing techniques to address this shortcoming.

Despite these advances, YOLOv8-FDA still faces several limitations: its training relies predominantly on the GWHD dataset, whose limited coverage of field environments, wheat varieties and developmental stages may reduce generalizability in heterogeneous scenarios; its 8.3GFLOPs computational footprint, while modest, must be further optimized to achieve genuine real-time performance on edge devices; and its applicability to other crops has yet to be validated.

Future research will therefore prioritize cross-scenario transfer learning-leveraging domain-adaptation and few-shot strategies to enhance robustness in novel environments-edge-deployment optimization through neural architecture search and quantization-aware training to drive computational demands below 5GFLOPs, and the construction of a universal agricultural detection framework by developing multi-crop joint training paradigms and evaluating transfer performance on tasks such as spike counting and disease identification, thereby advancing intelligent solutions for precision agriculture.

## 5 Conclusions

In response to the limitations of existing wheat ear detection and counting methods, this study proposes an improved YOLOv8-based model (YOLOv8-FDA), which significantly enhances the accuracy of wheat ear detection and counting in drone images. The integration of RFAConv, DySample, HWD, and SDL modules provides a comprehensive solution for handling small-scale targets, occlusions, and complex field scenarios. On the GWHD dataset, the improved model achieves precision, recall, and mAP@0.5 of 86.3%, 77.5%, and 84.9%, respectively, representing improvements of 1.4%, 3.3%, and 2.3% compared to the original YOLOv8n model. The parameter size and computational cost of the model are 2.96MB and 8.3GFLOPs, respectively. These results demonstrate the model’s strong performance and suitability for deployment on mobile devices in real-world agricultural environments.

Although all experiments were conducted on high-performance hardware (Intel i7-14700KF CPU and NVIDIA RTX 4070 Super GPU), the lightweight nature of YOLOv8-FDA (2.96 MB, 8.3 GFLOPs) ensures its feasibility for deployment on resource-constrained edge devices such as Jetson Nano (128-core Maxwell GPU, 4 GB RAM, 472 GFLOPS) and Raspberry Pi 4B (quad-core ARM Cortex-A72 CPU, up to 8 GB RAM). This makes it highly suitable for real-time applications in agricultural environments, where resource constraints are common.

Future work will explore transfer learning approaches to further enhance the model’s adaptability across diverse application scenarios. Additionally, we will conduct further field tests to validate the model’s performance under different environmental conditions and optimize it for wider deployment in agricultural practices.

## Data Availability

Publicly available datasets were analyzed in this study. This data can be found here: Zenodo at https://doi.org/10.5281/zenodo.5092309.
